# Calciphylaxis Mimicry in End-Stage Renal Disease: Acute Limb Ischemia From Calcific Peripheral Arterial Disease

**DOI:** 10.7759/cureus.109854

**Published:** 2026-05-28

**Authors:** Alireza Izadian Bidgoli, Erik Isayan, Yaroslav Buryk, Elizabeth Bobr, Shabnam Yazdanpanah

**Affiliations:** 1 Department of Internal Medicine, American University of the Caribbean School of Medicine, Cupecoy, SXM; 2 Department of Pulmonary and Critical Care, Jackson Memorial Hospital, Miami, USA

**Keywords:** acute limb ischemia, calcific peripheral arterial disease, calciphylaxis mimicry, end-stage renal disease, vascular calcification

## Abstract

Calciphylaxis is a life-threatening complication of end-stage renal disease (ESRD) characterized by painful necrotic skin lesions resulting from small-vessel (arteriolar) calcification, thrombosis, and subsequent ischemic tissue necrosis. However, not all ischemic lesions in dialysis patients represent calciphylaxis. We present a 44-year-old man with ESRD on hemodialysis who developed progressive bilateral digital necrosis initially suggestive of calciphylaxis, given severe hyperphosphatemia and secondary hyperparathyroidism. Lesion morphology demonstrated well-demarcated distal gangrene without retiform purpura or induration, features atypical for calciphylaxis. Multimodal evaluation revealed an alternative diagnosis. Radiographs demonstrated extensive medial arterial calcification, while noninvasive vascular testing showed markedly reduced ankle-brachial indices and abnormal pulse volume recordings. Duplex ultrasonography identified hemodynamically significant arterial disease, and magnetic resonance imaging confirmed soft-tissue nonviability without osteomyelitis. Collectively, these findings supported acute limb ischemia secondary to advanced calcific peripheral arterial disease rather than calciphylaxis. The patient was managed with a vascular-focused approach, including evaluation for revascularization and surgical planning. This case highlights a critical diagnostic pitfall in ESRD, where dialysis-associated vascular calcification may mimic calciphylaxis while representing macrovascular occlusive disease. Accurate differentiation through careful clinical assessment and multimodal vascular evaluation is essential, as management strategies and outcomes differ substantially.

## Introduction

Peripheral arterial disease (PAD) arises from systemic atherosclerosis, resulting in progressive arterial narrowing and diminished blood flow to the lower extremities [1]. Among patients with end-stage renal disease (ESRD), PAD is further complicated by medial arterial calcification associated with chronic kidney disease-mineral bone disorder (CKD-MBD), which accelerates vascular stiffness and increases the risk of critical limb ischemia and tissue loss [1]. Hyperphosphatemia and secondary hyperparathyroidism promote osteogenic transformation of vascular smooth muscle cells, resulting in calcium-phosphate deposition within the arterial media and progressive vascular rigidity. In dialysis-dependent individuals, ischemic symptoms frequently present atypically and may not clearly indicate classic macrovascular disease [1-3]. Patients with ESRD and PAD experience substantially higher rates of cardiovascular mortality, limb loss, and major adverse vascular events compared with the general PAD population, underscoring the importance of early recognition and accurate diagnosis in this high-risk group [4].

Calciphylaxis, also known as calcific uremic arteriolopathy, represents a major diagnostic consideration in this population [1-3]. This rare but life-threatening condition is characterized by painful ischemic skin lesions resulting from small-vessel (arteriolar) calcification, thrombosis, intimal fibrosis, and subsequent tissue necrosis [2]. Patients with ESRD undergoing long-term dialysis frequently exhibit overlapping risk factors for both calciphylaxis and advanced PAD, including hyperphosphatemia, secondary hyperparathyroidism, diabetes mellitus, and extensive vascular calcification [1,2]. Consequently, painful necrotic skin lesions in these individuals are often difficult to attribute to a single etiology [2]. Mortality rates for calciphylaxis remain exceedingly high, emphasizing the importance of prompt recognition and early intervention [3].

This overlap creates a significant diagnostic challenge, as lesion morphology, clinical progression, and imaging findings may not reliably distinguish small-vessel calciphylaxis from macrovascular ischemia [2,3]. Misdiagnosis can delay appropriate treatment or lead to unnecessary interventions [3]. In the present case, chronic dialysis dependence and severe metabolic abnormalities initially raised concern for calciphylaxis despite objective evidence of advanced large-vessel occlusive disease.

This case is presented to highlight the diagnostic complexity of ischemic limb lesions in dialysis-dependent patients and to emphasize the importance of distinguishing calciphylaxis from calcific peripheral arterial disease in order to guide timely and appropriate management.

## Case presentation

Clinical presentation

A 44-year-old man with ESRD secondary to obstructive uropathy, status post-deceased-donor kidney transplant in 2020, complicated by graft failure and subsequent dialysis dependence, presented with approximately two weeks of progressively worsening bilateral lower extremity pain, most severe in the right foot, accompanied by progressive necrotic changes involving the toes. His medical history was notable for hypertension, diabetes mellitus, anemia of CKD, bilateral nephrectomy, secondary hyperparathyroidism, severe hyperphosphatemia, prior peritoneal dialysis catheter placement, and recent hospitalization for peritonitis, pneumonia, and empyema. He had recently completed or remained on broad-spectrum antimicrobial therapy and was readmitted with persistent systemic inflammatory findings, severe limb pain, and concern for worsening ischemic tissue loss.

Initial assessment and physical exam

On initial evaluation, the patient appeared acutely ill and required intensive monitoring. He was tachycardic and intermittently febrile, with preserved oxygenation and no focal neurologic deficits. Cardiopulmonary examination was notable for tachycardia without overt respiratory distress.

Examination of the lower extremities revealed ischemic changes involving multiple digits bilaterally. There was dry gangrene of the right hallux and distal aspects of the second and third toes, with well-demarcated black eschar and absence of purulence. Similar ischemic lesions were noted on the contralateral foot, including necrotic changes and areas of ulceration. The affected digits were cool to the touch with diminished or absent distal pulses and delayed capillary refill, consistent with compromised arterial perfusion. Additional findings included localized necrotic lesions over the heels. The overall pattern of tissue injury was distal, well-demarcated, and noninflammatory in appearance, favoring ischemic necrosis rather than an infectious or vasculitic process (Figure 1).

**Figure 1:**
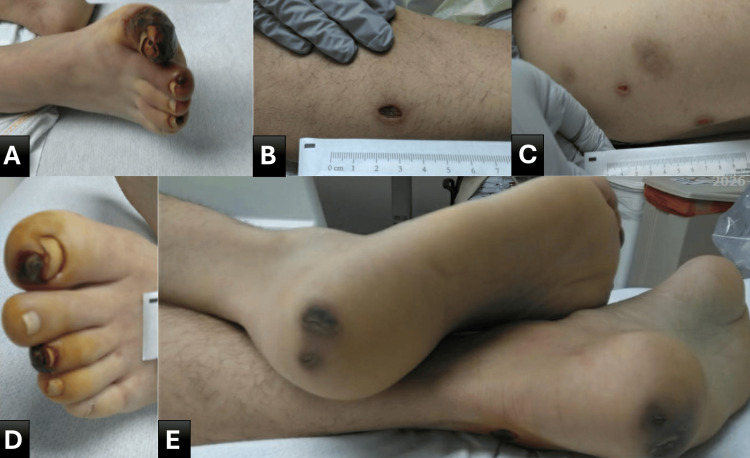
Clinical photographs demonstrating ischemic tissue necrosis (A) Dry gangrenous necrosis of the right hallux and distal toes with black eschar formation and ischemic discoloration. (B) Ulcerative necrotic lesion of the lower extremity with central eschar. (C) Multiple ulcerative skin lesions involving the lower extremities. (D) Dry gangrenous necrosis involving the left distal toes with ischemic discoloration and eschar formation. (E) Bilateral heel eschars and ischemic ulcerations consistent with advanced peripheral ischemic injury in the setting of severe calcific peripheral arterial disease

Importantly, the morphology and distribution of the lesions were not characteristic of calciphylaxis, as there was an absence of retiform purpura, widespread livedo racemosa, or intensely painful indurated plaques with progressive ulceration. Instead, the findings were more consistent with acute-on-chronic limb ischemia in the setting of severe peripheral arterial disease and dialysis-associated vascular calcification.

Laboratory and diagnostic testing

Initial laboratory studies demonstrated marked leukocytosis, with white blood cell counts exceeding 19,000-22,000/µL and neutrophil predominance, consistent with systemic inflammation or infection. Hemoglobin was severely reduced, ranging from approximately 6.5 to 8.0 g/dL during the admission, requiring multiple packed red blood cell transfusions, with transfusions initiated for hemoglobin levels below 7.0 g/dL. Platelets were markedly elevated earlier in the course, above 700,000-800,000/µL, consistent with reactive thrombocytosis, though later values varied. Chemistry studies were consistent with ESRD, with creatinine values ranging from approximately 5.6 to >10 mg/dL and severely reduced estimated glomerular filtration rate. Hyperphosphatemia was prominent, with phosphorus values around 9-10 mg/dL, and the patient had known secondary hyperparathyroidism treated with cinacalcet and phosphate binders. Inflammatory markers were elevated, including C-reactive protein greater than 45 mg/L. Cardiac markers showed troponin elevation felt to represent type II myocardial injury in the setting of tachycardia, sepsis physiology, anemia, and renal disease. Table 1 summarizes this patient's laboratory results at admission and during hospitalization.

**Table 1:** Summary of laboratory findings at admission and during hospitalization in a patient with end-stage renal disease and acute limb ischemia Admission values represent the earliest documented laboratory measurements upon presentation, while the range reflects the minimum and maximum values recorded during the hospital course. Reference ranges are institutionally standardized adult values and are provided with corresponding units. Abnormal values are interpreted in the context of the patient’s underlying ESRD, systemic inflammatory state, and acute illness. Variability in platelet count reflects a dynamic clinical course, including an initial reactive thrombocytosis followed by subsequent thrombocytopenia. Elevated inflammatory markers, persistent leukocytosis, and derangements in renal function are consistent with severe systemic illness in the setting of chronic kidney disease and suspected infection NT-proBNP: N-terminal pro-B-type natriuretic peptide

Parameter	Admission value	Range during hospitalization	Reference range (unit)
White blood cell count	19.7	16.9-22.6	4.0-11.0 × 10⁹/L
Hemoglobin	7.5	6.5-8.0	13.5-17.5 g/dL
Hematocrit	25.3	22-26	41-53%
Red blood cell count	2.93	2.5-3.1	4.5-5.9 × 10¹²/L
Mean corpuscular volume	86.3	83-88	80-100 fL
Mean corpuscular hemoglobin	25.9	25-26	27-33 pg
Mean corpuscular hemoglobin concentration	30.0	28-31	32-36 g/dL
Red cell distribution width	17.2	17.0-18.4	11.5-14.5%
Platelet count	730	678-814	150-400 × 10⁹/L
Sodium	138	135-140	135-145 mmol/L
Potassium	4.0	3.8-4.6	3.5-5.0 mmol/L
Chloride	101	96-101	98-106 mmol/L
Bicarbonate	24	20-27	22-29 mmol/L
Blood urea nitrogen	31	31-75	7-20 mg/dL
Creatinine	5.60	5.6-10.49	0.6-1.3 mg/dL
Glucose	133	108-172	70-100 mg/dL
Phosphorus	9.0	9.0-10.2	2.5-4.5 mg/dL
Albumin	2.6	2.3-3.0	3.5-5.0 g/dL
C-reactive protein	>45	Persistently >45 mg/L	<5 mg/L
Ferritin	3,250	3,250	30-400 ng/mL
Troponin I	0.474	0.474-0.568	<0.04 ng/mL
NT-proBNP	59,900	59,900	<125 pg/mL
Osmolality (calculated)	294	294-309	275-295 mOsm/kg
Anion gap	13	13-23	8-16 mmol/L

Blood gas analysis demonstrated no severe acid-base disturbance, with venous pH ranging 7.41-7.42, partial pressure of carbon dioxide 35-44 mmHg, partial pressure of oxygen 39-43 mmHg, and bicarbonate 22-27 mEq/L. Microbiologic testing revealed molecular blood culture detection of *Staphylococcus epidermidis* with identification of the mecA/C resistance gene, raising concern for methicillin-resistant coagulase-negative staphylococcal bacteremia vs. contamination. Other organisms, including *Staphylococcus aureus*, Enterococcus species, *Streptococcus pneumoniae*, and major Gram-negative pathogens, were not detected. Peritoneal fluid analysis demonstrated clear ascitic/peritoneal fluid with low nucleated cell counts and no evidence of active infection, arguing against ongoing peritonitis during this admission. Methicillin-resistant *Staphylococcus aureus* nasal polymerase chain reaction testing was negative (Table 2).

**Table 2:** Microbiologic and peritoneal diagnostic findings This table summarizes microbiologic and peritoneal diagnostic testing obtained during hospitalization. Molecular blood culture testing identified *Staphylococcus epidermidis* with detection of the mecA/C resistance gene, raising concern for methicillin-resistant coagulase-negative staphylococcal bacteremia vs. contamination in the appropriate clinical context. Broad microbiologic testing was otherwise negative for major Gram-positive and Gram-negative pathogens. Peritoneal fluid analysis did not support peritonitis, and MRSA nasal screening was negative MRSA: methicillin-resistant *Staphylococcus aureus*; PCR: polymerase chain reaction

Category	Parameter	Result	Interpretation
Blood culture (molecular panel)	Staphylococcus epidermidis	Detected	Possible contaminant vs. true bacteremia (coagulase-negative Staphylococcus)
	mecA/C (methicillin resistance gene)	Detected	Suggests methicillin resistance if the organism is clinically significant
	Staphylococcus aureus	Not detected	No evidence of *Staphylococcus aureus* bacteremia
	Enterococcus faecalis/Enterococcus faecium	Not detected	No enterococcal bacteremia
	Streptococcus pneumoniae	Not detected	No pneumococcal bacteremia
	Gram-negative organisms panel	Not detected	No major Gram-negative bacteremia identified
Peritoneal fluid analysis	Appearance	Clear	Not suggestive of infection
	Nucleated cells	Low	No inflammatory response
	Culture	Negative	No evidence of peritonitis
MRSA screening	Nasal PCR	Negative	Low likelihood of MRSA colonization

Plain radiographs of the bilateral feet showed no acute fracture, dislocation, or significant soft-tissue swelling, but demonstrated severe vascular calcifications and bilateral calcaneal spurs (Figure 2).

**Figure 2:**
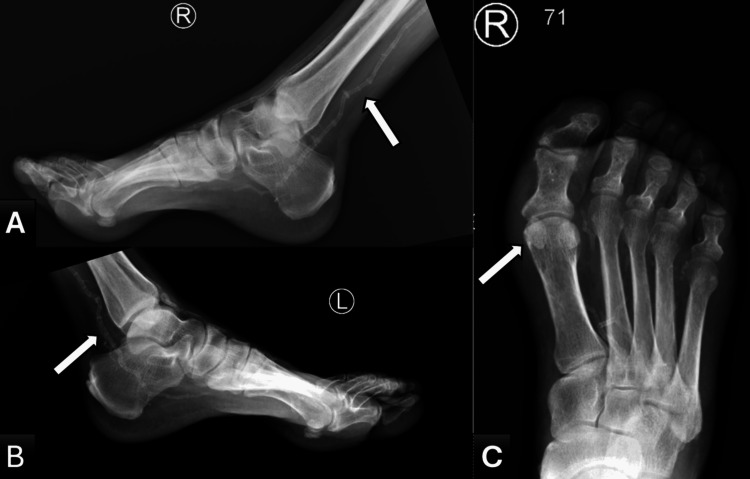
Radiographic evidence of extensive medial arterial calcification in the lower extremities (A) Lateral radiograph of the right foot demonstrating prominent linear calcifications along the arterial distribution (white arrow), consistent with medial arterial calcification. (B) Lateral radiograph of the left foot showing similar diffuse vascular calcifications involving distal arterial structures (white arrow). (C) Anteroposterior radiograph of the right foot demonstrating additional arterial calcifications and associated osseous changes (white arrow), without radiographic evidence of osteomyelitis. These findings are characteristic of advanced vascular calcification in the setting of end-stage renal disease and support a macrovascular etiology of limb ischemia rather than calciphylaxis

Bilateral lower extremity arterial duplex ultrasonography demonstrated hemodynamically significant disease, including markedly elevated peak systolic velocity in the right mid superficial femoral artery (376.7 cm/s), consistent with high-grade stenosis (>75% diameter reduction), along with evidence of severe distal arterial insufficiency characterized by dampened spectral waveforms and reduced flow in the posterior tibial artery (Figure 3 and Table 3). Segmental pressure measurements and pulse volume recordings (PVRs) further revealed markedly abnormal hemodynamics, with reduced ankle-brachial indices (ABI) bilaterally (right 0.46, left 0.57) and progressive distal waveform attenuation, indicating severe multilevel arterial disease (Figure 4). These findings, in conjunction with imaging evidence of diffuse vascular calcification, supported a diagnosis of advanced peripheral arterial disease leading to acute limb ischemia.

**Figure 3:**
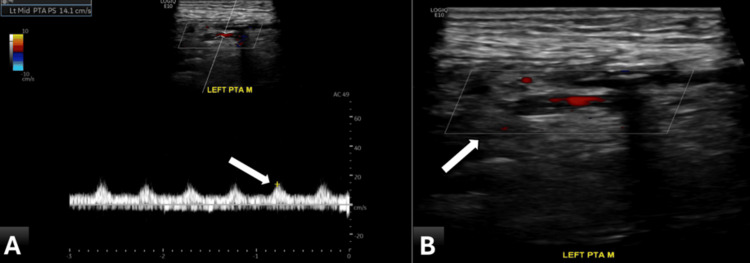
Duplex ultrasonography demonstrating distal arterial flow impairment in the posterior tibial artery (A) Spectral Doppler waveform obtained from the left posterior tibial artery demonstrating markedly reduced peak systolic velocity (~14 cm/s) with dampened waveform morphology (white arrow), consistent with severe distal arterial insufficiency. (B) Color Doppler ultrasound image of the left posterior tibial artery showing diminished and heterogeneous intraluminal flow (white arrow), reflecting impaired perfusion in the setting of advanced peripheral arterial disease

**Table 3:** Duplex ultrasound velocity mapping demonstrating hemodynamically significant multilevel arterial disease Duplex ultrasonography velocity measurements of bilateral lower extremity arteries demonstrating abnormal flow dynamics across multiple vascular segments. Notably, there is markedly elevated peak systolic velocity in the right mid superficial femoral artery (376.7 cm/s), consistent with hemodynamically significant stenosis. Distal vessels, including the posterior tibial and dorsalis pedis arteries, demonstrate significantly reduced velocities, reflecting impaired distal perfusion. These findings confirm multilevel arterial disease with both focal high-grade stenosis and downstream flow limitation, consistent with advanced peripheral arterial disease PS: peak systolic velocity; AC: angle correction; EIA: external iliac artery; CFA: common femoral artery; DFA: deep femoral artery; SFA: superficial femoral artery; Pop A: popliteal artery; ATA: anterior tibial artery; PTA: posterior tibial artery; DPA: dorsalis pedis artery

Vessel	Right PS (cm/s)	Right AC (deg)	Left PS (cm/s)	Left AC (deg)
EIA	62.4	43	41.9	11
CFA	50.6	43	71.6	44
DFA	59.8	43	62.5	49
Prox SFA	50.9	50	84.8	49
Mid SFA	376.7	50	75.0	49
Dist SFA	61.2	50	49.3	49
Pop A	36.4	50	54.4	49
Prox ATA	36.4	50	33.4	49
Dist ATA	18.3	50	35.1	49
Prox PTA	52.0	50	34.2	49
Dist PTA	6.3	50	23.4	49
DPA	11.9	50	14.2	49

**Figure 4:**
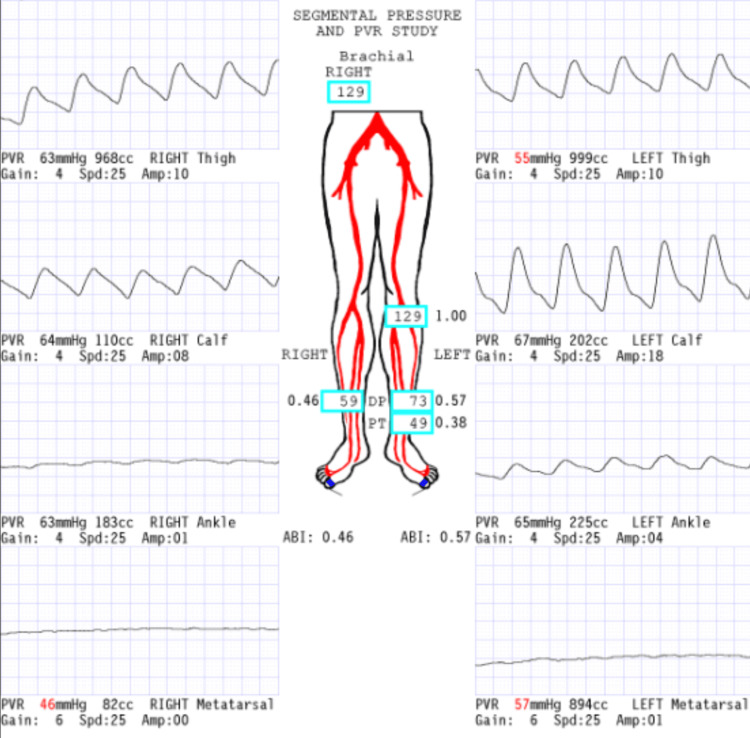
Segmental pressure and pulse volume recording analysis demonstrating severe bilateral peripheral arterial disease Segmental pressure measurements and PVRs of the lower extremities demonstrate markedly abnormal hemodynamics. ABI are significantly reduced bilaterally (right ABI: 0.46, left ABI: 0.57), consistent with moderate-to-severe peripheral arterial disease. Progressive waveform dampening and loss of amplitude are observed distally, particularly at the ankle and metatarsal levels, indicating severe distal perfusion impairment. These findings support advanced macrovascular occlusive disease contributing to critical limb ischemia PVRs: pulse volume recordings; ABI: ankle-brachial indices

MRI of the right foot performed with and without intravenous contrast demonstrates gangrenous changes involving the right hallux extending to the level of the proximal interphalangeal joint (Figure 5). There is focal hypoenhancement in the plantar aspect of the great toe consistent with nonviable soft tissue. No marrow signal abnormalities or enhancement are identified to suggest osteomyelitis. Surrounding soft-tissue structures, including tendons and plantar fascia, appear grossly intact without evidence of abscess or deep space infection. These findings support ischemic tissue necrosis in the setting of severe peripheral arterial disease rather than an infectious etiology.

**Figure 5:**
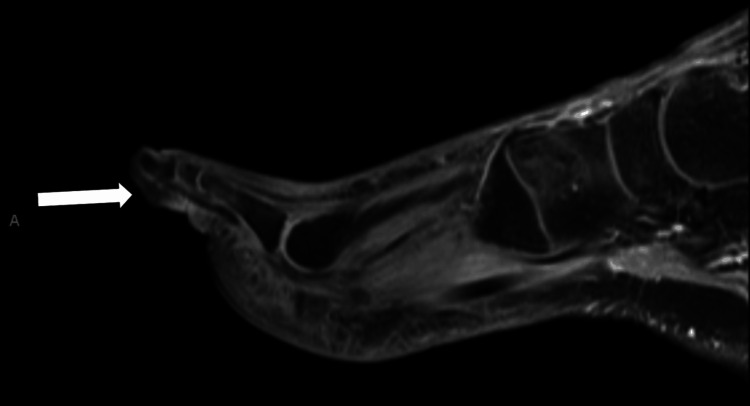
Magnetic resonance imaging demonstrating soft-tissue nonviability without osteomyelitis Sagittal MRI of the foot demonstrating areas of soft-tissue signal abnormality and nonviable tissue involving the distal foot (white arrow), without radiographic evidence of cortical destruction or marrow edema to suggest osteomyelitis

Computed tomography (CT) angiography of the chest and CT of the abdomen/pelvis with contrast were performed to evaluate for pulmonary embolism, infection, and possible thoracic mass. The study showed no pulmonary embolism at the lobar pulmonary arterial level.

A major incidental but clinically important CT finding was a large high-attenuation mass centered along the lateral aspect of the left seventh rib, associated with osseous erosion and pathologic fracture of the adjacent rib, suspicious for malignancy (Figure 6). An additional partially calcified periarticular mass was noted adjacent to the left acromion, further highlighting the extent of extraskeletal calcific disease.

**Figure 6:**
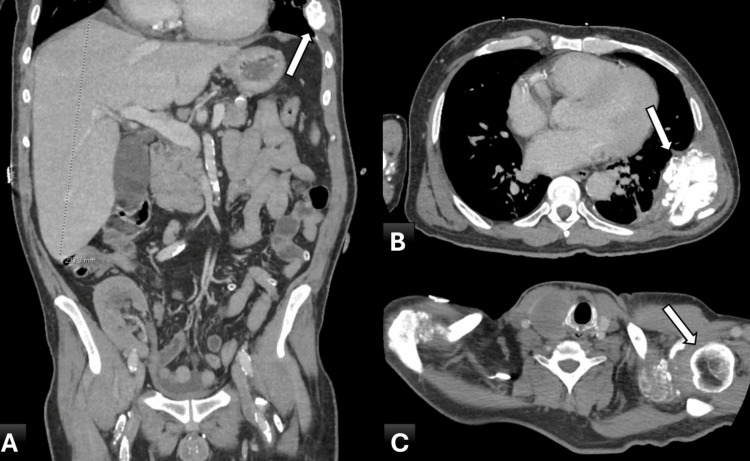
Incidental calcified chest wall mass with osseous involvement on computed tomography (A) Coronal CT image demonstrating a large high-attenuation mass along the lateral aspect of the left chest wall centered at the level of the seventh rib, with associated osseous erosion and pathologic fracture (white arrow). (B) Axial CT image of the thorax showing a densely calcified soft-tissue mass adjacent to the ribs (white arrow), corresponding to the chest wall lesion. (C) Axial CT image at the level of the shoulder demonstrating an additional partially calcified periarticular mass adjacent to the left acromion (white arrow). These findings represent incidental mass-like calcified lesions with associated osseous involvement in the setting of advanced systemic calcific disease, raising concern for underlying neoplastic versus dystrophic calcific processes CT: computed tomography

Multidisciplinary board discussion

The case was reviewed by critical care, nephrology, vascular surgery, podiatry, infectious disease, endocrinology, ENT, interventional radiology, and surgical teams. The central diagnostic issue was whether the patient’s lower extremity necrosis represented calciphylaxis or ischemic gangrene from severe calcific peripheral arterial disease. Although ESRD, hyperphosphatemia, and secondary hyperparathyroidism created a strong clinical background for calciphylaxis, the pattern of disease was more consistent with macrovascular occlusive disease: severe arterial calcification on radiographs and CT, abnormal ABI/PVR studies, focal right superficial femoral artery stenosis, distal waveform abnormalities, and MRI-confirmed nonviable toe tissue without osteomyelitis. Subsequent documentation noted that the biopsy did not support calciphylaxis and instead showed findings consistent with acquired perforating dermatosis (perforating collagenosis), a condition associated with CKD but not directly related to the ischemic limb presentation.

Vascular surgery concluded that the patient had severe peripheral arterial disease with acute-on-chronic limb ischemia and dry gangrene, but no immediate vascular intervention was pursued while he remained medically unstable and septic. Podiatry agreed that the gangrenous digits were stable and dry, without evidence of acute purulent infection, and that amputation would likely be required once medically optimized. Infectious disease evaluated the possibility of persistent infection from recent peritonitis, pneumonia, empyema, vascular access, or necrotic tissue, and continued antimicrobial management. Nephrology transitioned dialysis planning from peritoneal dialysis toward hemodialysis and addressed severe mineral-bone disease. ENT and endocrinology evaluated the large right thyroid mass with tracheal deviation, while oncology and orthopedic oncology evaluation were recommended for the destructive rib/acromial lesions suspicious for malignancy.

Diagnosis and management

The patient’s presentation initially raised concern for calciphylaxis given his ESRD, hyperphosphatemia, and secondary hyperparathyroidism. However, lesion morphology, absence of characteristic skin findings, and nonsupportive histopathology made this diagnosis unlikely. Instead, noninvasive vascular testing, radiographic evidence of extensive arterial calcification, and MRI confirmed tissue nonviability supported advanced peripheral arterial disease as the cause of acute limb ischemia.

Clinical outcome

During hospitalization, the patient remained medically complex but had stabilized sufficiently to be downgraded from the intensive care unit to the medical floor. His ischemic toe lesions remained dry and demarcated, with no MRI evidence of osteomyelitis or of a clear purulent infection. No emergent vascular intervention was performed during the unstable phase, and the plan was continued medical optimization followed by definitive vascular or surgical management as appropriate. The case highlights a diagnostically important presentation of acute limb ischemia in a dialysis patient caused by severe calcific peripheral arterial disease, mimicking calciphylaxis but ultimately better explained by large-vessel calcification and macrovascular ischemia.

## Discussion

Background****

Peripheral artery disease (PAD) is a manifestation of systemic atherosclerosis, characterized by stenosis or occlusion of arteries supplying the lower extremities [5]. Early clinical descriptions of intermittent claudication from the 19th century established the foundation for the current understanding of PAD as a systemic vascular disorder rather than a localized limb disease [5]. Contemporary literature shows that PAD in CKD and ESRD constitutes a distinct and particularly severe form, associated with significantly higher morbidity [5]. Although PAD largely affects older adults, it presents at a younger age and progresses more rapidly in ESRD populations [6].

PAD affects over 200 million people worldwide, with prevalence increasing with age [5]. Among patients with ESRD, prevalence estimates range from 25% to 40%, with even higher rates observed in dialysis populations [5]. PAD is associated with markedly increased risks of cardiovascular events, limb ischemia, and mortality, especially in people with advanced kidney disease [5,6].

Risk factors for PAD in ESRD include diabetes mellitus, hypertension, hyperlipidemia, and smoking, all of which are highly prevalent in this population [6]. Additionally, CKD-specific mechanisms such as uremia, chronic inflammation, oxidative stress, and disturbances in mineral metabolism, particularly vascular calcification, play a central role in accelerating atherosclerosis and promoting more distal, calcific disease [5,6]. PAD most frequently involves the lower extremity arteries, especially the femoral, popliteal, and infrapopliteal vessels [6]. In ESRD, the disease more commonly affects smaller, distal vessels and is often more diffuse and calcified [5,6].

PAD in ESRD has similarities with calciphylaxis, a disease defined by painful ischemic necrotic lesions in patients with ESRD [7]. Clinical context, lesion location, and specific diagnostic tests are essential for distinguishing calciphylaxis from PAD and related conditions. A key differentiating feature is the presence of small-vessel calcification and a netlike pattern of calcification on plain radiographs in calciphylaxis, findings that are absent in PAD [7]. Table 4 highlights the key similarities and differences between PAD and calciphylaxis secondary to ESRD [1-3,5-7].

**Table 4:** Comparative clinical and diagnostic features of calciphylaxis and peripheral arterial disease in end-stage renal disease This table highlights overlapping and distinguishing clinical, diagnostic, and pathophysiologic features of calciphylaxis and peripheral arterial disease in patients with ESRD ESRD: end-stage renal disease; CKD-MBD: chronic kidney disease-mineral and bone disorder; PTH: parathyroid hormone; ABI: ankle-brachial index; CTA: computed tomography angiography; MRA: magnetic resonance angiography; PAD: peripheral arterial disease

Feature	Calciphylaxis (in ESRD)	Peripheral arterial disease (PAD) in ESRD	Shared features in ESRD
Primary pathophysiology	Small-vessel (arteriolar) calcification and thrombosis causing cutaneous ischemia	Large- and medium-vessel atherosclerosis causing arterial narrowing/occlusion	CKD-related vascular calcification and mineral imbalance contribute to both
Typical pain pattern	Severe pain out of proportion to examination findings	Claudication progressing to rest pain in advanced disease	Painful lower extremity lesions may occur in both
Skin findings	Violaceous plaques/nodules progressing to necrotic ulcers/eschar	Cool, pale, shiny skin with hair loss; ulcers commonly involve toes or pressure areas	Ischemic skin ulcers and poor wound healing
Pulses	Often preserved	Diminished or absent distal pulses	-
Key clinical clue	Severe pain with necrotic skin lesions despite preserved pulses (microvascular disease)	Claudication/rest pain with diminished pulses and macrovascular occlusive disease	-
Risk factors	Hyperphosphatemia, elevated calcium-phosphate product, secondary hyperparathyroidism, warfarin use, obesity, diabetes, female sex	Diabetes, smoking, hypertension, dyslipidemia, advanced age, CKD-MBD, chronic inflammation	Common in patients with ESRD and diabetes
Diagnostic evaluation	Clinical suspicion with confirmatory skin biopsy showing arteriolar medial calcification	ABI, toe-brachial index, Doppler ultrasound, CTA/MRA, angiography	Diagnostic evaluation may be challenging in ESRD because vascular calcification can affect ABI accuracy
Histopathology	Medial calcification and thrombosis of dermal arterioles	Atherosclerotic plaque with macrovascular luminal stenosis/occlusion	Vascular calcification may be present in both
Treatment approach	Wound care, pain control, optimization of calcium/phosphate/PTH balance, sodium thiosulfate, discontinuation of triggering agents	Antiplatelet therapy, statins, risk-factor modification, wound care, revascularization when indicated	Both require aggressive wound management and infection prevention
Prognosis	High mortality, often related to infected necrotic wounds and sepsis	Increased cardiovascular morbidity, limb loss, and chronic limb-threatening ischemia	Elevated risk of amputation and mortality

Pathology/pathophysiology

Peripheral arterial disease in ESRD arises from accelerated systemic atherosclerosis in conjunction with uremia-specific vascular injury [5]. The principal cells involved in lesion development are vascular smooth muscle tissue (VSMT) and endothelial tissue [5]. CKD-MBD induces VSMT transformation into an osteogenic phenotype through disrupted calcium phosphate metabolism, resulting in medial arterial calcification and increased arterial stiffness [5].

​Endothelial dysfunction, resulting from decreased nitric oxide bioavailability, leads to oxidative stress and chronic inflammation, which promote lipid deposition and plaque formation [6]. In ESRD, the presence of uremic toxins and hyperphosphatemia further enhances proinflammatory signaling and vascular calcification pathways, thereby accelerating atherosclerosis beyond the impact of traditional risk factors [6,7].

​Progressive intimal plaque accumulation and medial calcification result in luminal narrowing and reduced perfusion of distal tissues [5,6]. These changes cause ischemia, which may manifest clinically as intermittent claudication or advance to chronic limb-threatening ischemia (CLTI) characterized by ulceration and tissue necrosis [6]. Histological examination reveals atherosclerotic plaques in the intima and medial calcification, features that distinguish ESRD-associated peripheral arterial disease (PAD) from typical PAD [5-7].

Comparative analysis with the current literature****

*Clinical Presentation* 

Vascular calcification is a well-recognized complication of ESRD, particularly among patients with prolonged dialysis exposure, diabetes mellitus, hyperphosphatemia, and secondary hyperparathyroidism [8]. Severe vascular calcification in CKD involves vascular smooth muscle cell osteogenic transformation rather than passive mineral deposition alone [8]. In contrast to classic calciphylaxis, which typically presents with painful retiform purpura, livedo reticularis, indurated plaques, and ulcerative skin lesions caused by calcification of dermal arterioles, CLTI presents with well-demarcated dry gangrene of the toes [9-11]. Although calciphylaxis remains an important diagnostic consideration in dialysis patients, atypical presentations should indicate evaluation of alternative differentials such as CLTI [9]. Most notably, our patient presented with CTLI at 44 years old, when this condition is typically seen in older patient populations [12].

*Diagnostic Workup* 

Distinguishing calciphylaxis from severe peripheral arterial disease in dialysis patients remains diagnostically challenging because both conditions may present with painful tissue necrosis [9]. Skin biopsy remains the diagnostic gold standard for calciphylaxis and typically demonstrates medial calcification of dermal arterioles, thrombosis, and fibrointimal hyperplasia [10]. In our case, biopsy findings did not support calciphylaxis, though skin biopsy cannot directly confirm macrovascular disease; the diagnosis of PAD was established through vascular imaging and hemodynamic studies. Radiographs revealed diffuse linear vascular calcifications consistent with medial arterial calcification commonly seen in ESRD patients [8]. Duplex ultrasonography demonstrated hemodynamically significant right superficial femoral artery stenosis, while ABI of 0.46 on the right and 0.57 on the left confirmed severe bilateral ischemia. MRI further excluded osteomyelitis while confirming soft-tissue nonviability, helping to distinguish ischemic gangrene from infectious causes. Recent literature emphasizes the importance of multimodal vascular imaging in dialysis patients with necrotic lesions to avoid misdiagnosing macrovascular ischemia as calciphylaxis [9-12]. Differential diagnoses in similar cases include vasculitis, diabetic foot infection, septic emboli, and thromboembolic disease [11].

*Management* 

Standard treatment of calciphylaxis typically includes aggressive phosphate control, intravenous sodium thiosulfate, wound care, pain management, and consideration of parathyroidectomy in refractory cases [9,10,13]. In contrast, management of CLTI focuses on restoring perfusion when feasible, infection control, and surgical treatment of nonviable tissue [11,12]. In this case, vascular intervention was deferred because the patient remained medically unstable. Broad-spectrum antimicrobial therapy was initiated because infection remained a competing concern during hospitalization. The patient also required a blood transfusion and optimization of dialysis management, including treatment of severe mineral bone disease contributing to ongoing vascular calcification. Planned delayed amputation after medical stabilization aligned with current CLTI recommendations for patients who are not immediate candidates for revascularization [11,13].

*Clinical Outcome* 

Patients with CLTI have high risks of limb loss, repeat interventions, and mortality, particularly in medically complex populations with significant comorbid disease burden such as ESRD [11-13]. Our patient stabilized sufficiently to be downgraded from the intensive care unit without progression to wet gangrene or osteomyelitis, representing a favorable short-term outcome despite ongoing long-term risk. Because long-term follow-up was limited, the ultimate risk of future amputation, recurrent ischemia, and mortality remains uncertain in this patient.

What we learned from this case 

This case illustrates that necrotic skin lesions in patients with ESRD are not synonymous with calciphylaxis and should prompt careful consideration of alternative etiologies, including macrovascular ischemia. Although the patient exhibited multiple metabolic risk factors strongly associated with calciphylaxis, including severe hyperphosphatemia, secondary hyperparathyroidism, and extensive vascular calcification, the ultimate diagnosis was acute-on-chronic limb ischemia caused by advanced calcific peripheral arterial disease. This distinction reflects fundamentally different pathophysiologic processes with markedly different therapeutic implications.

A key insight from this case is the importance of prioritizing lesion morphology, distribution, and hemodynamic assessment over metabolic risk factors alone. The sharply demarcated distal gangrene, absence of retiform purpura or indurated plaques, diminished distal perfusion, and objective vascular abnormalities were more consistent with macrovascular occlusive disease than calciphylaxis. This highlights the continued importance of careful clinical examination in an era increasingly reliant on laboratory and imaging data, and underscores how overreliance on risk profiles may contribute to diagnostic anchoring, a cognitive bias in which initial impressions disproportionately influence subsequent clinical decision-making.

This case also emphasizes the critical role of multimodal vascular evaluation in dialysis-dependent patients with necrotic lesions. Markedly reduced ABI, abnormal pulse-volume recordings, duplex ultrasonography demonstrating flow-limiting superficial femoral artery stenosis, and extensive medial arterial calcification collectively established the presence of severe hemodynamically significant peripheral arterial disease. These findings support the concept that dialysis-associated vascular calcification is not merely a passive radiographic phenomenon but may progress to clinically significant luminal compromise, critical limb ischemia, and tissue loss.

Equally important are the consequences of diagnostic misclassification. Calciphylaxis and macrovascular ischemia require fundamentally different management strategies: the former focuses on correcting metabolic derangements and sodium thiosulfate therapy, whereas the latter necessitates urgent vascular assessment, consideration of revascularization, and surgical management of nonviable tissue. Failure to distinguish between these entities may delay appropriate intervention, expose patients to ineffective therapies, and increase the risk of amputation and mortality.

More broadly, this case highlights the need to conceptualize vascular disease in ESRD as a spectrum rather than as isolated diagnostic entities. Microvascular calciphylaxis and macrovascular calcific peripheral arterial disease may coexist or clinically mimic one another, particularly in patients with advanced CKD-MBD. Careful integration of clinical findings, histopathology, vascular imaging, and hemodynamic data remains essential for accurate diagnosis and individualized management.

Ultimately, this case provides a clinically important perspective by demonstrating that severe dialysis-associated vascular calcification may masquerade as calciphylaxis while representing advanced macrovascular occlusive disease. Recognition of this distinction is essential to avoid diagnostic error and to facilitate timely, mechanism-directed management in a uniquely vulnerable patient population.

## Conclusions

This case describes a dialysis-dependent patient with ESRD who presented with progressive lower extremity necrosis initially concerning for calciphylaxis but ultimately found to have acute-on-chronic limb ischemia secondary to advanced calcific peripheral arterial disease. Despite overlapping metabolic risk factors, lesion morphology, absence of classic calciphylaxis features, and objective vascular testing supported a macrovascular etiology. Radiographic evidence of diffuse vascular and extraskeletal calcification further suggested a systemic calcific vasculopathy rather than isolated small-vessel disease.

The patient stabilized following multidisciplinary evaluation and medical optimization without emergent surgical intervention. This case highlights an important diagnostic pitfall, as calcific peripheral arterial disease may closely mimic calciphylaxis in dialysis-dependent patients. Distinguishing between these entities is critical given their differing pathophysiology, management strategies, and prognostic implications. Clinicians should maintain a broad differential diagnosis and integrate clinical morphology, vascular imaging, histopathology, and hemodynamic data to facilitate accurate diagnosis and timely intervention.
